# Autonomous robotic alignment and dynamic illumination control of an ophthalmic slit lamp for *in vivo* anterior segment imaging

**DOI:** 10.1117/1.JBO.31.12.123304

**Published:** 2026-07-22

**Authors:** Morgan McCloud, Nipun Hewage, Joseph A. Izatt, Mark Draelos, Anthony N. Kuo, Ryan P. McNabb

**Affiliations:** a Duke University, Department of Biomedical Engineering, Durham, North Carolina, United States; b Duke University Medical Center, Department of Ophthalmology, Durham, North Carolina, United States; c University of Michigan, Department of Robotics, Ann Arbor, Michigan, United States; d University of Michigan Health, Department of Ophthalmology, Ann Arbor, Michigan, United States

**Keywords:** slit lamp, illumination engineering, medical robots, ophthalmic imaging, automatic alignment

## Abstract

**Significance:**

The slit lamp is the gold standard ophthalmic instrument for examining the anterior segment of the eye. Slit lamps require the mechanical stabilization of patients to facilitate alignment and a specialized technician to operate. The growing demand for ophthalmic care and shortages of specialists highlight the need for alternative diagnostic technologies.

**Aim:**

We demonstrate an autonomously aligning, contactless slit lamp enabled by a vision-guided robot to acquire anterior segment images without mechanical patient stabilization.

**Approach:**

We integrate three subsystems mounted to a collaborative robot arm: a custom optical system for dynamic slit illumination, vision-based sensors, and color cameras. A hierarchical control strategy is employed to maintain alignment. Programmable illumination patterns are projected onto the cornea to replicate slit-lamp examination maneuvers.

**Results:**

The device autonomously aligned to the eyes of *in vivo* human subjects, and we collected color images of the anterior segment during alignment. Dynamic slit illumination compensated for motion with a latency of 86 ms, allowing slit alignment to be maintained during imaging without mechanical stabilization.

**Conclusions:**

These results demonstrate the feasibility of autonomous, contactless slit-lamp imaging through the use of robotic alignment and dynamic illumination. Such advancements could expand access to anterior segment diagnostic imaging.

## 1 Introduction

Patients frequently present to urgent and emergent care facilities seeking eye care.^1^^,^^2^ Therefore, it is imperative that the medical providers at these facilities have adequate instrumentation and training to image, diagnose, and/or refer patients in a timely manner. However, recent studies of emergency medicine physicians report generally low self-reported confidence in using ophthalmic equipment^3^ and managing ophthalmic emergencies.^4^ Furthermore, access to specialist eye care professionals remains limited in many regions, underscoring the need for alternative ophthalmic diagnostic imaging technologies.^5^^–^^7^

**Fig. 1:**
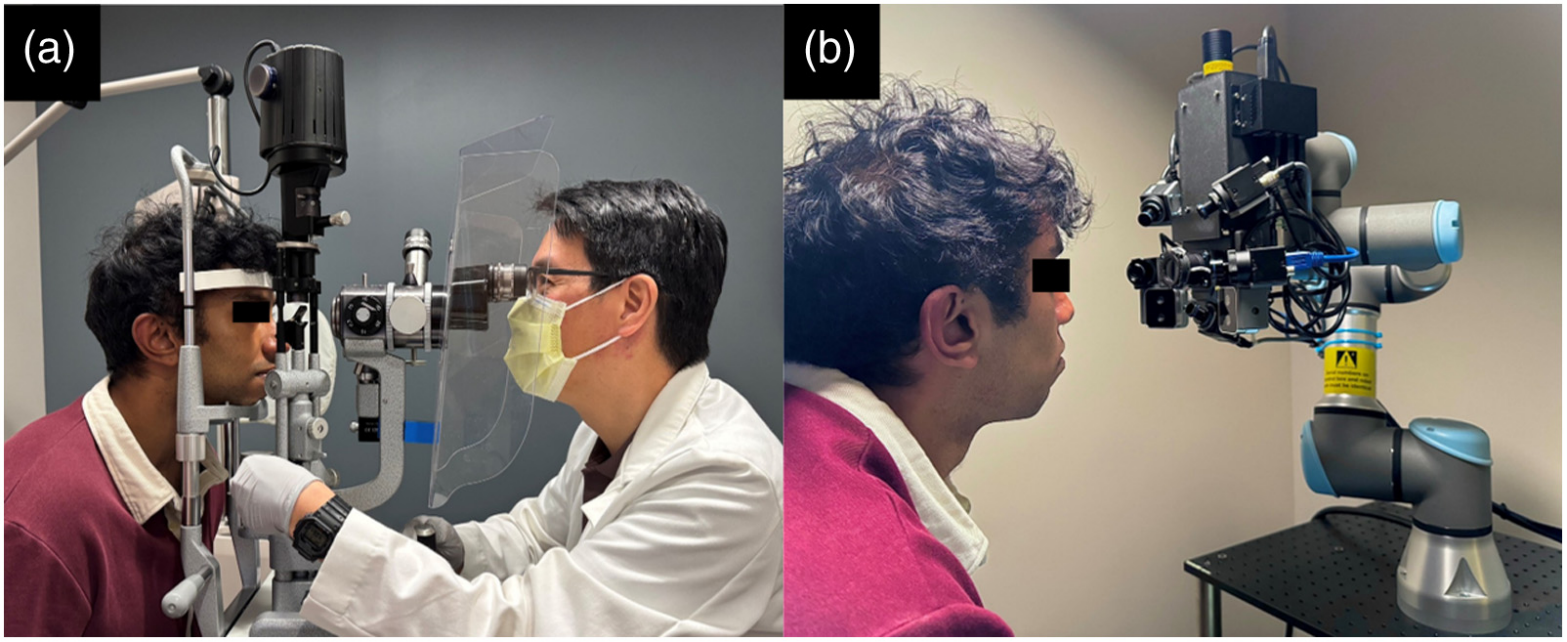
Comparison of slit-lamp examination using the traditional (a) and proposed robotic (b) approaches.

Most individuals who seek ocular care present with anterior ocular pathologies.^1^ The ophthalmic slit lamp is the clinical standard for examining the ocular anterior segment.^8^ During slit-lamp examinations, an examiner utilizes the device to project a variably sized slit of light onto the cornea to create an optical cross-section and observes the illuminated tissue through an integrated biomicroscope [Fig. 1(a)]. The standard slit-lamp exam requires that the patient is mechanically stabilized in the system with a chin and forehead rest. Certain populations of patients often have difficulty achieving and maintaining the posture required for the ophthalmic slit lamp. For example, individuals with limited mobility or larger bodies may experience discomfort or be unable to properly position within the device.

Recent developments in handheld and teleoperated ophthalmic imaging aim to reduce operator burden and increase imaging accessibility.^9^^,^^10^ Portable slit lamps have been developed, which often utilize smartphones for illumination and digital image capture.^11^^–^^13^ Although these systems are highly portable and improve usability relative to conventional slit lamps, these handheld devices still require fine alignment from both the photographer and patient to obtain clinically useful images of the anterior segment. A teleoperated motorized slit lamp has also been developed to expand ophthalmic screening to remote locations.^14^ However, this teleoperated system required patient stabilization and a remotely located operator to virtually interact with the system for alignment and photography of the subject.

To address these limitations, we developed an autonomously vision-guided robotic slit lamp consisting of (1) dynamic illumination optics, (2) dual high-resolution color cameras, and (3) patient motion tracking cameras in a single integrated end-effector mounted to a 6- degree-of-freedom collaborative robotic arm [Fig. 1(b)]. Unlike the previously described portable or teleoperated approaches, our system requires neither mechanical stabilization of the patient nor a highly trained operator. We achieve tracking and alignment of individuals without mechanical stabilization of the head or chin with a multistage approach to feature tracking utilizing robotic and optical compensation. Our robotic system detects the freely standing or sitting patient and aligns to their eye as opposed to the traditional ophthalmic imaging devices, in which the patient is brought to the device and stabilized for imaging. Like a traditional slit lamp, our device projects variably-sized slits of white light onto the cornea. The system’s robotic arm autonomously aligns the module to the cornea of a nonstabilized individual in a contactless manner. Dynamic illumination, decoupled from robotic motion, enables programmable light projection to replicate clinical slit-lamp maneuvers. Two high-resolution color cameras capture and store anterior segment slit-lamp data for subsequent review by an eye care specialist, analogous to commercial digital slit lamps.

Prior works have developed autonomously aligned robotic imaging devices toward more accessible diagnostic imaging for other major ophthalmic imaging modalities, such as optical coherence tomography (OCT)^15^^–^^19^ and retinal fundus photography.^20^ Using our robotic OCT methodology^21^ as a foundational platform, we extend our robotic imaging framework to the slit-lamp biomicroscope by integrating a slit illumination module and color imaging system onto a robotic manipulator. Unlike OCT, slit-lamp imaging introduces distinct challenges in anterior eye localization, structured illumination projection, and high-resolution color video acquisition, which we address in this work.

The remainder of this paper is organized as follows: We describe the optical design and methods of slit illumination and outline the mechanical design of the module mounted to the robot end-effector. We then detail the control algorithms used for robotic auto-alignment and dynamic slit illumination. In addition to outlining the specifications of the device, we demonstrate device feasibility through autonomous alignment and color video acquisition of multiple slit maneuvers on nonmechanically stabilized human (*in vivo*) participants and their eyes.

## 2 Methods

### 2.1 Slit Illumination Optical System Design

**Fig. 2:**
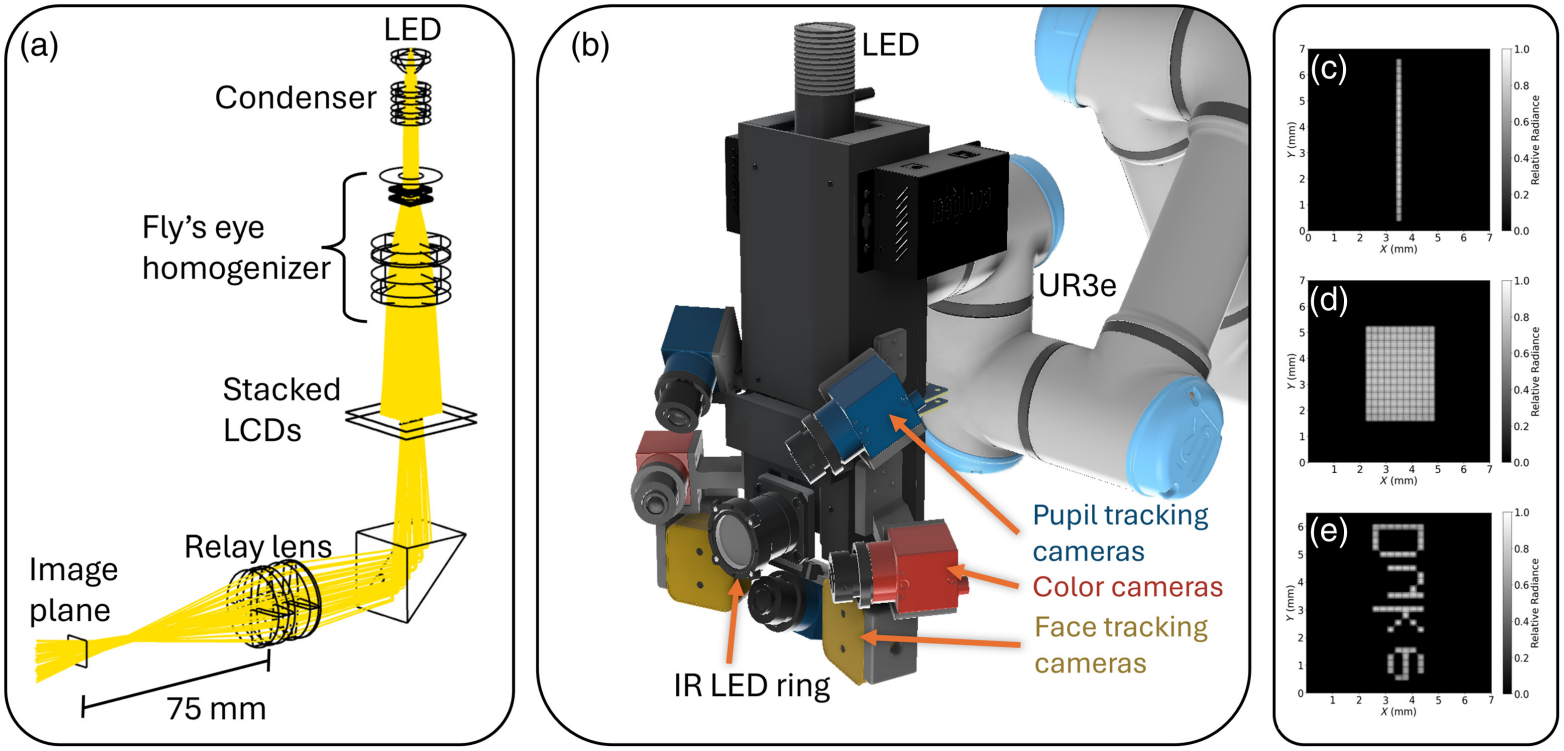
Optical and mechanical design of the slit-lamp end effector. (a) Ansys Zemax rendering of slit-lamp projection design. (b) Autodesk Fusion rendering of the mechanical mounting design. (c)–(e) Ansys Zemax simulation of optical system projection at eye (image plane) with LCDs configured to project (c) a slit, (d) a rectangle, and (e) the word “Duke” assuming perfect contrast between on and off pixels on the LCDs. The right-angle prism and relay result in a vertical flip in the image plane.

The optical system [Fig. 2(a)] was designed to generate a position-programmable slit profile on the eye while maintaining a compact and lightweight form factor suitable for robotic mounting. We designed and optimized the optical system for slit projection in Ansys Zemax in nonsequential mode. The light source is a current-controlled, nonpolarized white LED (4900 K, Thorlabs MNWHL4, Newton, New Jersey, United States). The LED output is first collimated via a condensing lens and directed through a fly’s eye homogenizer. The fly’s eye homogenizer consists of a pair of microlens arrays and a collecting lens, which results in a homogeneous top-hat intensity profile.^22^^,^^23^ The specifications of the imaging optics can be found in Table S1 in the Supplementary Material.

Conventional slit lamps utilize Köhler illumination to produce a uniform output onto the eye.^24^ By contrast, the fly’s eye configuration reduces optical path length and overall weight due to the compact focal lengths of microlens arrays, facilitating miniaturization for robotic mounting. The top-hat profile from the fly’s eye homogenizer was imaged onto two stacked transmissive LCD panels (Display Visions EA-DOGM128, Gilching, Germany), which functioned as a programmable slit aperture. Simultaneous pixel-level control of the LCD stack facilitated dynamic modulation of slit shape and position [Fig. 2(c)–2(e)]. Stacking two LCD panels improved the effective contrast relative to a single transmissive display. We relayed the programmed aperture onto the eye with a working distance of 75 mm. The optical system was characterized by analyzing the projected slit at the image plane. The output was imaged directly onto a CMOS sensor (Sony IMX183, Tokyo, Japan, for distortion measurements, ON Semi PYTHON 1300, Phoenix, Arizona, United States, for homogeneity measurements) to quantify slit size and geometric distortion. The minimum achievable spot size was limited by the size and subsequent magnification of a single pixel of the LCD at the image plane. Minimum spot size was defined as the full width at half maximum intensity of the illumination profile (FWHM). We also measured the 10% to 90% edge width across a transition between dark and light portions at the image plane. The illumination depth of field was evaluated by measuring FWHM and 10% to 90% edge width over a 3.2 mm range. Local geometric distortion, which identifies pincushion or barrel distortion effects, was measured following ISO 17850:2015.^25^ Distortion of a grid point i is defined as

$$D_{i}=\frac{H_{i}^{*}-H_{i}}{H_{i}}\times 100,$$ (1)

where $H_{i}^{*}$ is the measured distance of point i from the center of an expanded grid at the image plane and $H_{i}$ is the expected distance. Distortion was measured by sequentially activating individual pixels across a 15 by 15 grid and calculating centroid positions at the image plane to determine $H_{i}^{*}$. Expected centroid spacing ($H_{i}$) was computed from the measured LCD pixel size at the image plane.

The homogeneity of the slit was evaluated using beam uniformity metrics defined by ISO 13694:2018,^26^ where beam uniformity is the normalized root mean square deviation of the beam energy above a fractional clip level η,

$$U_{\eta }=\frac{1}{E_{\eta ,\mathrm{avg}}}\sqrt{\frac{1}{A_{\eta }^{i}}\iint [E(x,y)-E_{\eta ,\mathrm{avg}}{]}^{2}dx\;dy.}$$ (2)

$E_{\eta ,\mathrm{avg}}$ and $A_{\eta }^{i}$ denote the mean energy density and irradiation area above the threshold $E_{\max}*\eta$.

Michelson contrast was evaluated by measuring optical power with all LCD pixels activated and deactivated. Algorithms and intermediate measurements for distortion, homogeneity, and contrast can be found in the Supplemental Material.

### 2.2 Hardware and Mechanical Device Design

The device required the illumination optics, tracking cameras, and color imaging subsystems to be rigidly mounted into a single end-effector assembly for robotic slit alignment and imaging [Fig. 2(c)]. The full module was designed to remain within the mass payload limits of the collaborative robot arm while preserving the clinical geometry of conventional slit-lamp imaging, wherein the slit illumination is observed at an angular offset.

We designed custom opto-mechanical mounts (Autodesk Fusion), based on optic positions exported from Ansys Zemax, for attachment to the end-effector of a collaborative robot arm (Universal Robots, UR3e). The total mass of the integrated end-effector assembly was 1.3 kg, remaining below the UR3e payload limit of 3 kg (Fig. 2).

Color imaging cameras were positioned with a ±30  deg offset from the optical axis of the slit projection to enable cross-sectional visualization of illuminated anterior chamber structures (Teledyne FLIR, BFS-U3-200S6C-C, Wilsonville, Oregon, United States). Two RGB-D cameras (Intel, Realsense D405, Santa Clara, California, United States) mounted on either side of the relay lens were used for face tracking, stage one of the robot alignment process. Three monochromatic cameras (Teledyne FLIR BFS-U3-16S2M-CS) mounted to the sides and below the relay lens captured data for pupil tracking, stage two of the robot alignment process (see Sec. 2.3). The pupil tracking cameras were filtered to capture infrared images of the eye, which resulted in high iris-pupil contrast independent of visible iris pigmentation.^27^ A ring of 940 nm LEDs around the relay lens provided illumination for the pupil tracking cameras.

### 2.3 Robot and Dynamic Illumination Control Algorithms

**Fig. 3:**
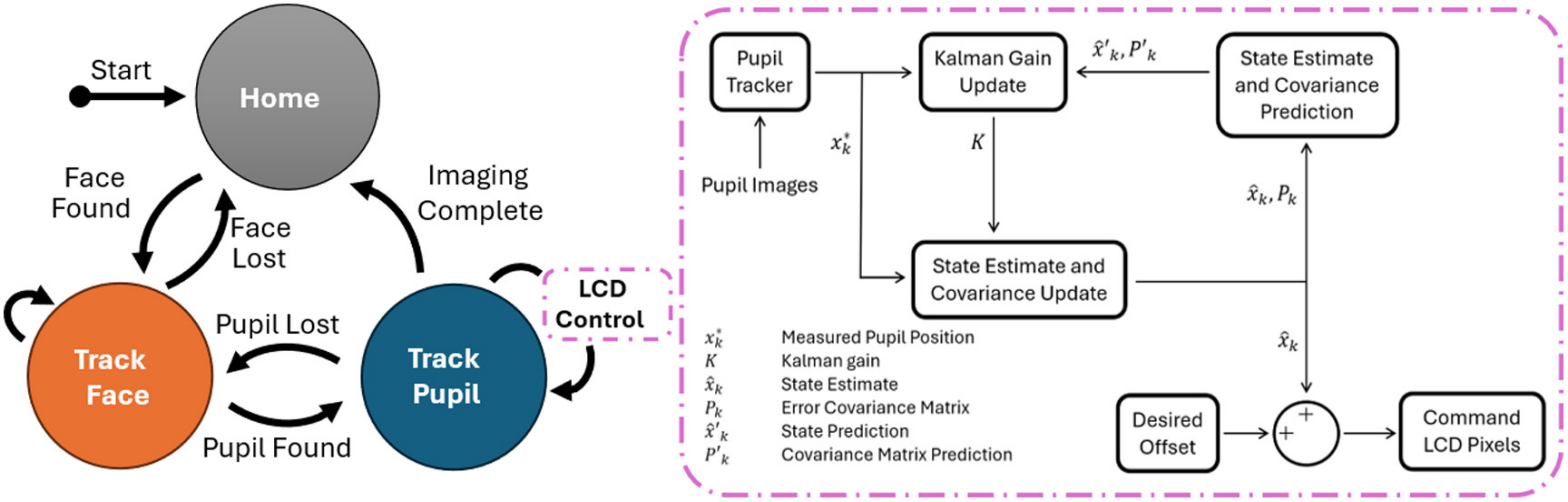
State transition diagram for robotic control and Kalman-filtered slit predictive control diagram for robotic alignment and small eye motion compensation.

Autonomous slit-lamp imaging of freely standing or sitting individuals requires active compensation of large-amplitude head motion and fine ocular motion, all while maintaining safe robotic operation. To address the challenges of multiscale alignment, we implemented a hierarchical control schema consisting of coarse face tracking-based alignment, fine pupil triangulation alignment, and optical projection refinement utilizing programmable illumination (Fig. 3). We adapted face tracking and pupil tracking stages previously developed for robotically guided ophthalmic optical coherence tomography systems.^15^^,^^21^^,^^28^

**Fig. 4:**
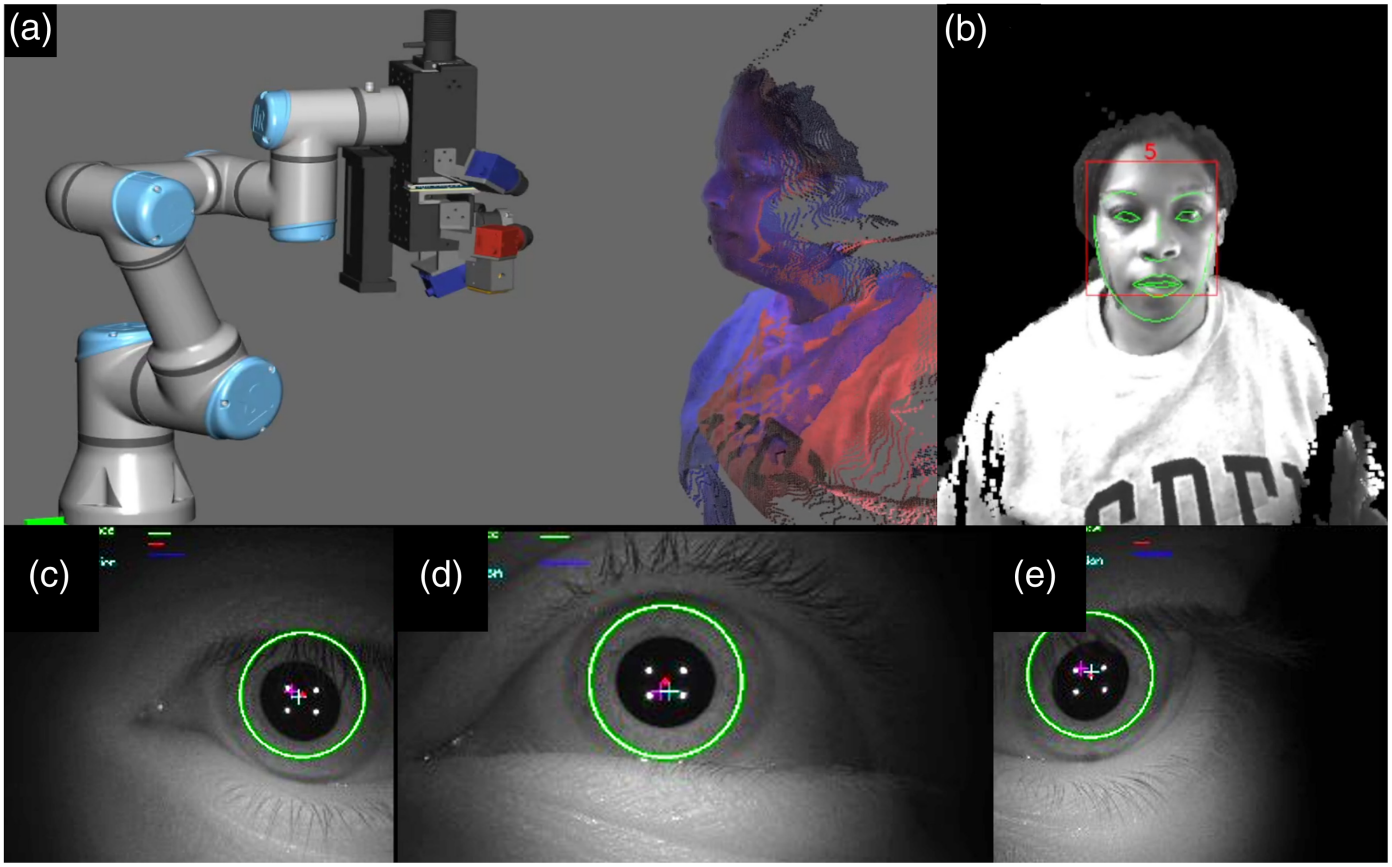
Tracking information obtained by pupil and face tracking sensors. (a) Real-time simulated robot workspace with face tracking point cloud. (b) Composite *en face* frame of face/eye segmentation from the perspective of the robot end-effector. Left (c), lower (d), and right (e) pupil camera views with segmented pupil centroid positions (red cross) and reprojected pupil center from triangulated position (cyan cross).

To initiate an imaging session, a subject sat or stood in front of the robot, which began parked in the “home” position. The two RGB-D face tracking cameras generated point clouds, which were composited to generate a 3D scene and transformed to match the end-effector coordinate frame. The 3D scene was then used to localize and segment facial landmarks utilizing the dlib C++ toolkit^29^ (Fig. 4). The 3D position of a selected eye was acquired by mapping the segmented landmark back to the 3D point cloud. A time-optimal Cartesian trajectory was generated from the home position to the 3D position of the eye^21^ such that the eye was in view of the pupil tracking cameras. It is notable that we utilize two depth cameras where, theoretically, a single depth camera can be used to locate objects in Cartesian space. However, when the robot approaches the subject with only a single camera, occlusions of facial features required for consistent segmentation can occur (e.g., the nose obstructing portions of the face). To mitigate this, we position two depth cameras on either side and slightly below the objective lens. This configuration enables more reliable and smoothly integrated 3D point cloud generation of the head during alignment [Figs. 4(a) and 4(b)]. We defined the workspace of face tracking by measuring the region over which facial landmarks could be reliably segmented and reached from the robot’s home configuration. Face tracking and path planning were updated at a rate of 15 Hz.

Pupil tracking, the second stage of robot alignment, occurred once the robot followed the motion plan as defined by face tracking and was aligned to the target eye. The three monochromatic cameras were synchronized at a capture rate of 100 FPS. Pupil segmentation was performed in parallel using custom C++ software. The minimum number of 2D sensors required to perform 3D triangulation is two. We utilize three cameras for this task to incorporate redundancy into our tracking sensors to guarantee that a minimum number of cameras will have a clear view of the pupil, despite different facial features. Previous iterations of our robot-guided pupil tracking algorithms estimated pupil position from binarized images where the algorithm selected the centroid of the largest connected component with an aspect ratio of approximately 1:1.^15^^,^^28^ The 3D pupil position was then estimated by triangulating the found centroid positions from each camera. The binarization approach worked well for robotically aligned optical coherence tomography, where subjects were placed in a dark environment, and the pupil was naturally dilated and comparatively large within the image. However, when the eye was illuminated with visible, white light projected from the slit-lamp system, the constrictive pupillary response resulted in small connected component features relative to other dark regions in the image, such as eyelashes. In addition, varying levels of visible illumination may change the magnitude of pupil response while the robot is aligned to the eye, resulting in a binarized threshold value that no longer results in sufficient pupil detection.

**Fig. 5:**
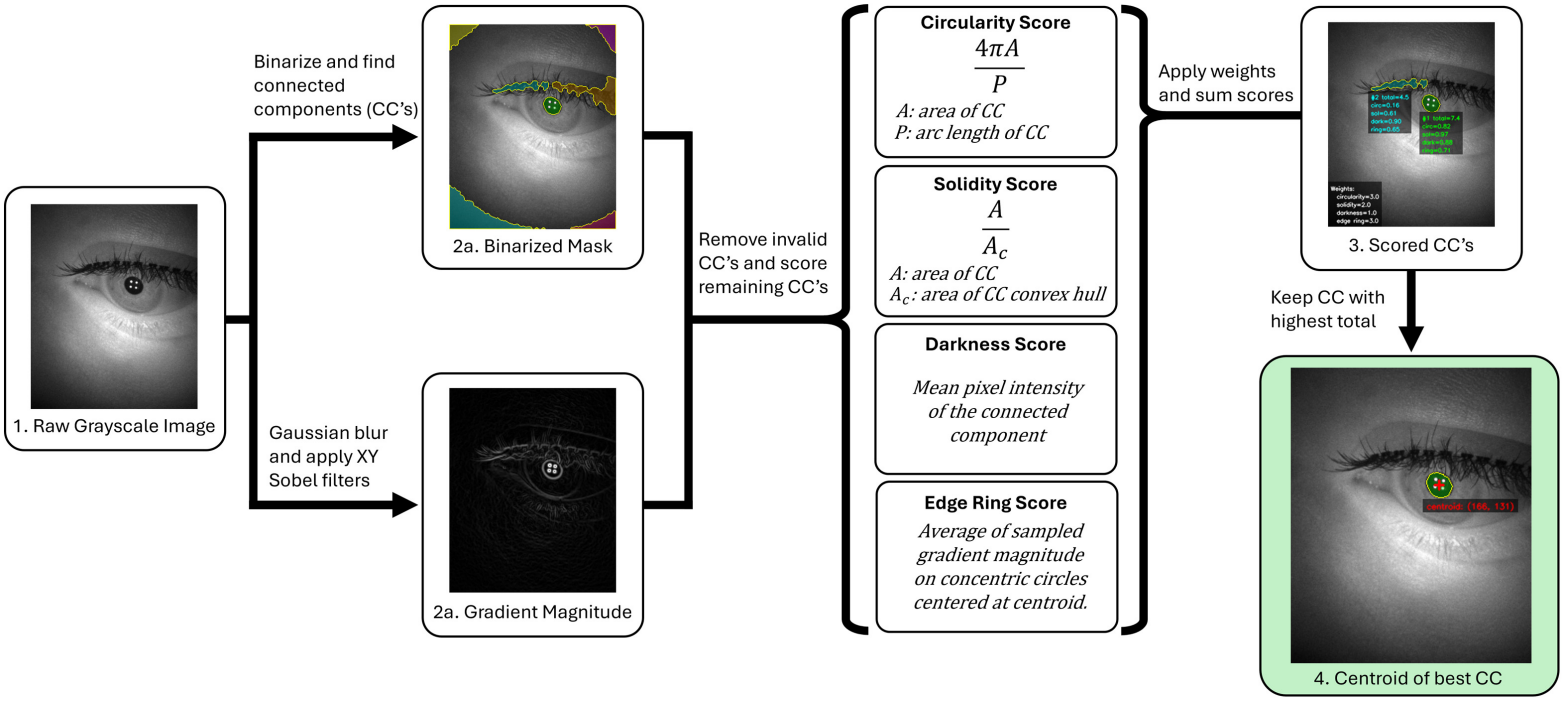
Flowchart of pupil segmentation algorithm with results of intermediate steps overlaid on an image captured of a healthy eye with one of the system’s pupil tracking cameras.

Given the dynamic illumination present in a slit-lamp system, we enhanced our pupil segmentation algorithm by introducing a quality score to connected component detection while maintaining a 100 FPS processing rate (Fig. 5). The new algorithm utilizes the rejection filters from previous iterations of pupil segmentation,^15^^,^^28^ including minimum size, maximum aspect ratio, and exclusion of pixels at the boundary of the image. However, instead of choosing the largest remaining connected component, ours identifies the pupil based on a weighted sum of four characteristics: circularity, solidity, darkness, and edge ring score. Pupils in infrared images tend to be highly circular dark components that present high contrast next to regions around it; therefore, those components are scored highly. The solidity score aims to discourage the algorithm from choosing the eyelashes as those can be larger, valid components when a nonmydriatic pupil naturally constricts when illuminated with visible light.

The highest score connected component was then defined as the pupil centroid, and each centroid from the three cameras is linearly triangulated to estimate 3D pupil position. If the pupil is lost after 200 pupil tracking cycles, the robot falls back to the face tracking stage, the binarization threshold will be stepped for each camera, and the threshold that results in the best connected component score will be utilized for a given pupil tracking camera. A minimum score must be obtained for a camera to consider the connected component as the true pupil to discourage thresholds that would identify the pupil but would ultimately be unstable over time. The pupil tracking cameras were stereo calibrated to one another and hand-eye calibrated to the robot end-effector with standard calibration techniques.^30^^,^^31^ To test pupil tracking accuracy and precision, a 5 mm pupil phantom was placed on a linear stage in view of the pupil tracking cameras and stepped in 0.5 mm increments for a total of 5 mm travel in each of the cardinal directions while the robot was stationary. The workspace of pupil tracking was measured by noting the full extent over which the pupil phantom could be accurately segmented.

Due to safety-imposed velocity limits on the robot (see Sec. 2.5), high-frequency pupil motion cannot be fully compensated through mechanical motion alone. Therefore, fine and rapid slit positioning was achieved through programmable modulation of the LCD aperture. The triangulated pupil position was fed to the LCD control loop, where a standard Kalman filter^32^ was utilized under a constant-velocity motion model to enable predictive slit placement (Fig. 3). Independent filters were used for the x and y directions. The process and measurement noise parameters of the Kalman filter were tuned empirically to $\sigma_{a}=15\;\mathrm{mm}\;s^{-1}$ and $\sigma_{b}=0.05\;\mathrm{mm}$, respectively.

For slit shapes that need to be in a specific position relative to the pupil, we applied an offset to the predicted center position. For the sweeping slit maneuver (see Sec. 2.5), this offset was ramped linearly over time to position ±3  mm at 1  mm/s around the center of the pupil. The LCD control loop update frequency was equivalent to pupil triangulation (100 Hz). The propagation delay of dynamic illumination reaction relative to pupil movement was evaluated as a measure of latency in LCD movement compensation. To characterize and measure this, a 5 mm pupil phantom was translated in 2 mm increments along a linear stage while pupil tracking and dynamic LCD illumination were activated. Pupil tracking estimates were treated as reference measurements for latency analysis, as the measured localization error was below the minimum achievable optical spot size of the projected slit.

### 2.4 Color Imaging

The color imaging subsystem was designed to enable visualization of corneal structure under slit illumination. The human cornea is ∼0.5  mm^33^^,^^34^ thick, and the anterior chamber depth is typically 3 to 4 mm.^35^^,^^36^ Therefore, the imaging system was designed to achieve a lateral resolution on the order of tens of micrometers across a millimeter-scale field of view and depth of field. Mechanical constraints associated with robotic mounting, including payload limits and center of gravity considerations of the end-effector assembly, required a short working distance and compact camera form factor.

The two angularly offset color imaging cameras mounted to the robot captured color video of the anterior segment at 10FPS (Teledyne FLIR, BFS-U3-200S6C-C). The color cameras utilized 25 mm, f/#5.6 S-mount lenses with an infrared cut filter to block light used for pupil tracking (Edmund Optics 24-472, Barrington, New Jersey, United States). The CMOS sensor contains 2.4  μm pixels but are binned 2× during imaging, resulting in an effective pixel size of 4.8  μm. When Nyquist sampling, the CMOS can theoretically resolve 9.6  μm structures. The camera lenses can focus to an Airy diameter of 2.44*λ*F, where λ is the wavelength of light imaged and F is the f/# of the imaging lens.^37^ A spectrogram of our LED illumination was measured, and the centroid of the spectrum was used for λ (Ocean Optics SR4 VIS-NR Spectrometer, Orlando, Florida, United States). The resulting Airy diameter was 7.74  μm, indicating that our imaging system is camera-limited while binning. Color images were captured at 1.5 MP (1440×1080  pixels), resulting in a focused field of view of 24.8×18.6  mm. The system’s spatial resolution and depth of field were quantified by imaging a USAF 1951 target at multiple points along the optical axis of the illumination subsystem. When imaging the resolution target, we only considered group-element pairs with a Michelson contrast of at least 10%.

### 2.5 Clinical Imaging and Safety Protocol

We demonstrated the system’s capabilities by imaging three healthy volunteers and one Duke Eye Center patient. The eye clinic patient was pharmacologically dilated as part of their clinical care before imaging by the robotic system. During an imaging session, the subject sat or stood in front of the robot. The operator initiated imaging and, once face detection was confirmed, selected the target eye via the graphical interface. The robotic slit-lamp system automatically aligned to the desired eye. The operator then selected the slit-lamp maneuver to perform and capture with the color cameras to demonstrate different dynamic illuminations: a stationary slit, full illumination, a small spot, or the word “Duke.” For all subjects, each maneuver was performed on both eyes. A sweeping slit was additionally utilized for the patient with pathology. For all maneuvers, except full illumination, we ramped LED power for three seconds to allow pupil tracking to accommodate any changes to pupil size. For full illumination, the LED was flashed for 1 s. The operator interface required no specialized ophthalmic imaging training.

All human subject research was conducted under a protocol approved by the Duke University Health System Institutional Review Board. Prior to imaging, informed consent was obtained from all participants. All portions of this research followed the tenets of the Declaration of Helsinki. Each given projection and scan maneuver utilized a different power level to ensure comfort for the subject while simultaneously conforming to the light exposure standards below those set by group 1 continuous wave instruments in ANSI Z80.36-2021.

Subjects were provided with an emergency stop button connected directly to the robot safety controller. If the safety controller detected an elbow or end-effector speed of >200  mm/s, the safeguard stop would trigger. Robot joint velocity limits for motion planning during each alignment stage are defined in Table 1. Linear end-effector velocities were evaluated during an imaging session of a healthy volunteer [Fig. 7(c)]. Applying the Jacobian across the corners of the robot’s manipulability polytope to find worst-case joint velocity limits^38^ revealed a theoretical linear velocity maximum of 706  mm/s. The percentage of this normalized velocity directed toward the subject varies based on where the subject chooses to stand in front of the system, but it was found for the evaluated session that this value was 80.6%. However, our control software never exceeded the safety bound of 200  mm/s during any imaging sessions. If a force of 40 N was encountered on the integrated force detector on the robot end-effector, an immediate software-initiated stop would trigger, though this safety feature was never activated during the imaging sessions.

**Table 1:** Robot joint velocity limits for different alignment stages.

	Joint velocity (rad/s)	Joint acceleration ($\mathrm{rad}/s^{2}$)	Joint jerk ($\mathrm{rad}/s^{3}$)
Home	45π/180	45π/180	$10^{4}\pi /180$
Face tracking	30π/180	30π/180	$10^{4}\pi /360$
Pupil tracking	45 π/180	30 π/180	$10^{4}\pi /180$

## 3 Results

### 3.1 Slit Illumination Specifications

**Fig. 6:**
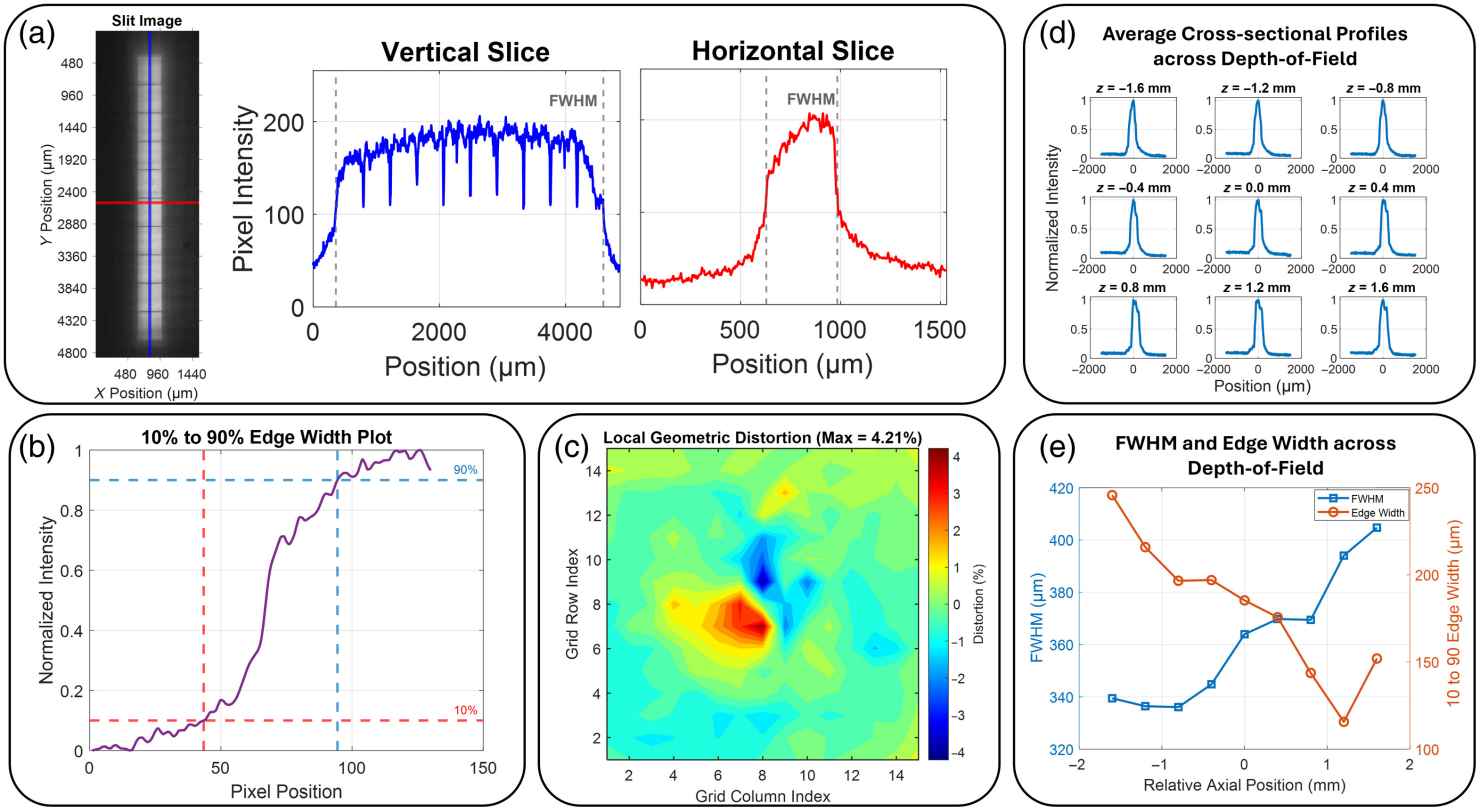
Slit illumination quality metric data. (a) Image of slit projected directly onto CMOS sensor with vertical (blue) and horizontal (red) cross sections of intensity projections. (b) 10% to 9% edge-width plot at a dark-bright transition. (c) Local geometric distortion measurements for a 15×15 projected LCD pixel grid at the image plane. (d) Cross-sectional horizontal profiles of slit illumination at multiple positions. (e) Slit illumination full width at half maximum (FWHM) and 10% to 90% edge width measured across multiple axial positions.

We assessed the slit illumination by measuring projected feature size, beam homogeneity, contrast, edge sharpness, and distortion at the image plane (Fig. 6). At the image plane, individual LCD pixels were projected with dimensions of 358×396  μm, whereas the maximum projected aperture measured 11.5×12.8  mm. We measured beam homogeneity to be 8.2% (where ideal is 0%) at η=0.7. We measured Michelson contrast to be 87.1%, and the 10% to 90% edge width was 140  μm. Maximum local geometric distortion was 4.21% across a 5.4×6.0  mm field of view at the image plane. We found that across a depth of field of 3.2 mm, the full width at half maximum (FWHM) of the slit illumination ranged from 336 to 405  μm, and the 10% to 90% edge width ranged from 116 to 246  μm [Fig. 6(e)]. Notably, the axial location that corresponded to the minimal FWHM was not the same as the minimal edge-width in either system, indicating that these metrics predict different optimal working distances. Small distortions across the field suggest that illumination patterns that span horizontally in the image plane will experience similar degradation over the depth of field. We measured slit illumination power of a clinical slit lamp (Haag-Streit BQ900, Köniz, Switzerland) and found that a slit of comparable size to our system, 3 mm wide, produced 710.2  μW through a 3.5 mm aperture. In comparison, our device illuminated the same power meter and sensor (Thorlabs PM100D and S120C) with a measured power of 251.2  μW.

### 3.2 Tracking Performance

We characterized the tracking capabilities of autonomous alignment by evaluating the operational workspace of face and pupil tracking, the accuracy and precision of pupil tracking, and the latency of predictive slit alignment. Face tracking could detect and align to eyes within a 360×180×210  mm volume centered ∼70  mm away from the final objective of the system. Pupil triangulation was achievable within a 35×42×52  mm volume centered 75 mm from the final objective. The x, y, and z pupil tracking mean accuracy and mean precision from incrementally stepping a pupil phantom on a linear stage are summarized in Table 2.

**Table 2:** Pupil tracking accuracy and precision summary.

Cartesian axis	Mean accuracy (μm)	Mean precision (1σ) (μm)
x	51.29	1.28
y	57.49	1.11
z	23.19	1.77

**Fig. 7:**
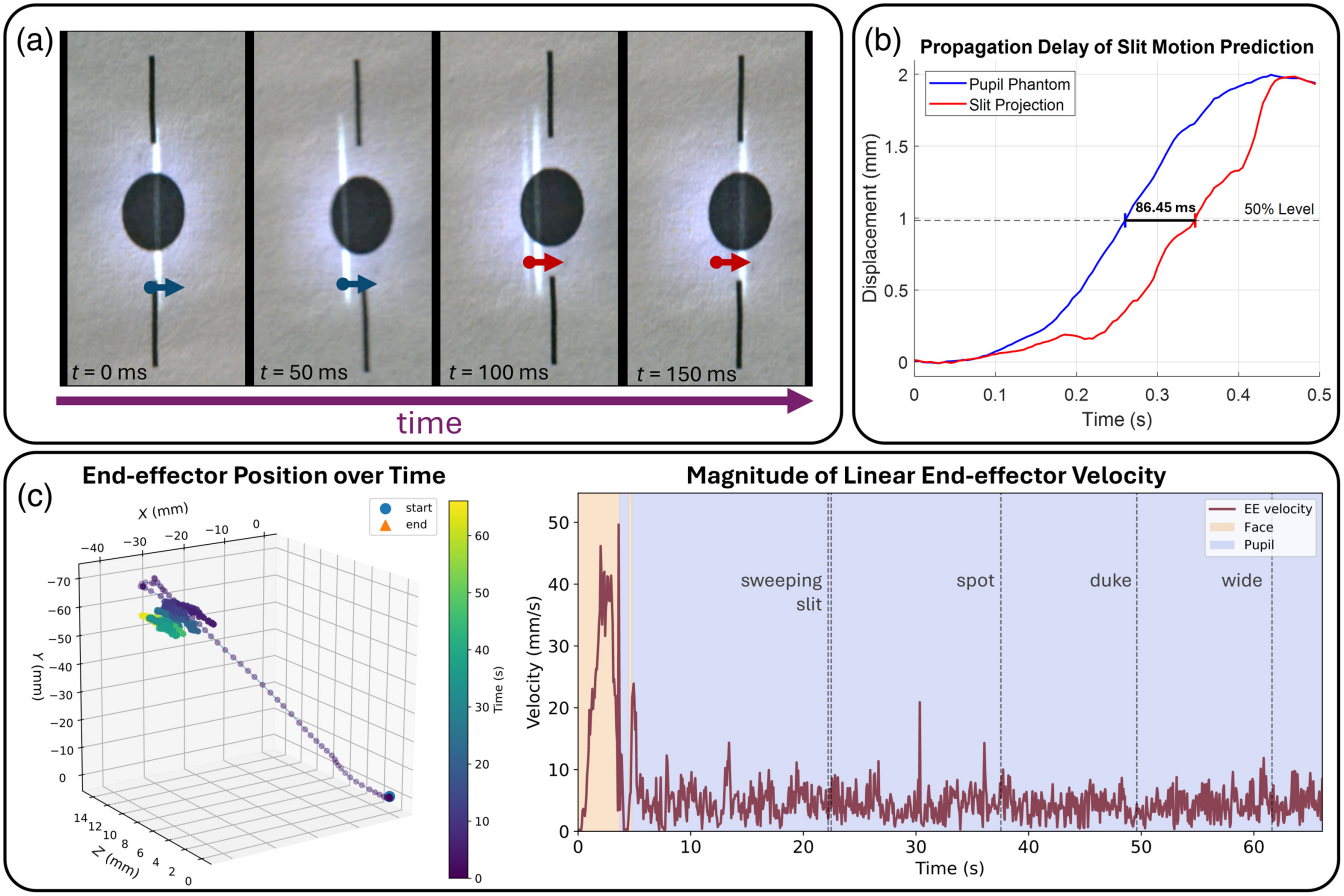
(a) Example of LCD slit compensation on a pupil phantom. At time zero, the slit begins to translate laterally (blue arrow), and after some latency, the slit is updated to the center of the pupil (red arrows). (b) Propagation delay plot of the position of a laterally translating pupil phantom and reaction from predictive slit illumination. (c) Tracked robot end-effector (EE) position and linear velocity while imaging a healthy volunteer. Times at which different illumination patterns were projected on the eye are marked with a gray dotted line.

Robot motion characteristics were evaluated during an imaging session of a healthy volunteer [Fig. 7(c)]. During face tracking and pupil tracking, the robot maintained a mean velocity of 22.5±14.4 and 4.5±2.7  mm/s, respectively. The session completed in 66.0 s and, during which, the robot reached a maximum velocity of 49.6  mm/s, well below the 200  mm/s safety limit. The latency of predictive slit illumination was evaluated by translating a pupil phantom while robot correction was inactive and LCD correction was enabled. The 50% propagation delay of slit illumination realignment was measured to be 86.45 ms [Figs. 7(a) and 7(b)]. At the color camera acquisition rate of 10FPS, this latency corresponds to just under the exposure time of one frame, which indicated slit realignment compensated for small motion within one or two frames. In this experiment, the pupil phantom translated with a mean velocity of 4.4  mm/s. In comparison, drift in a normal human eye during fixation generally occurs at <2  deg/s^39^ and corresponds to a lateral pupil speed of 0.42  mm/s, assuming an eye radius of 12 mm. This indicates that the system response is adequate for adjusting to drift during fixation.

### 3.3 Color Imaging Specifications

**Fig. 8:**
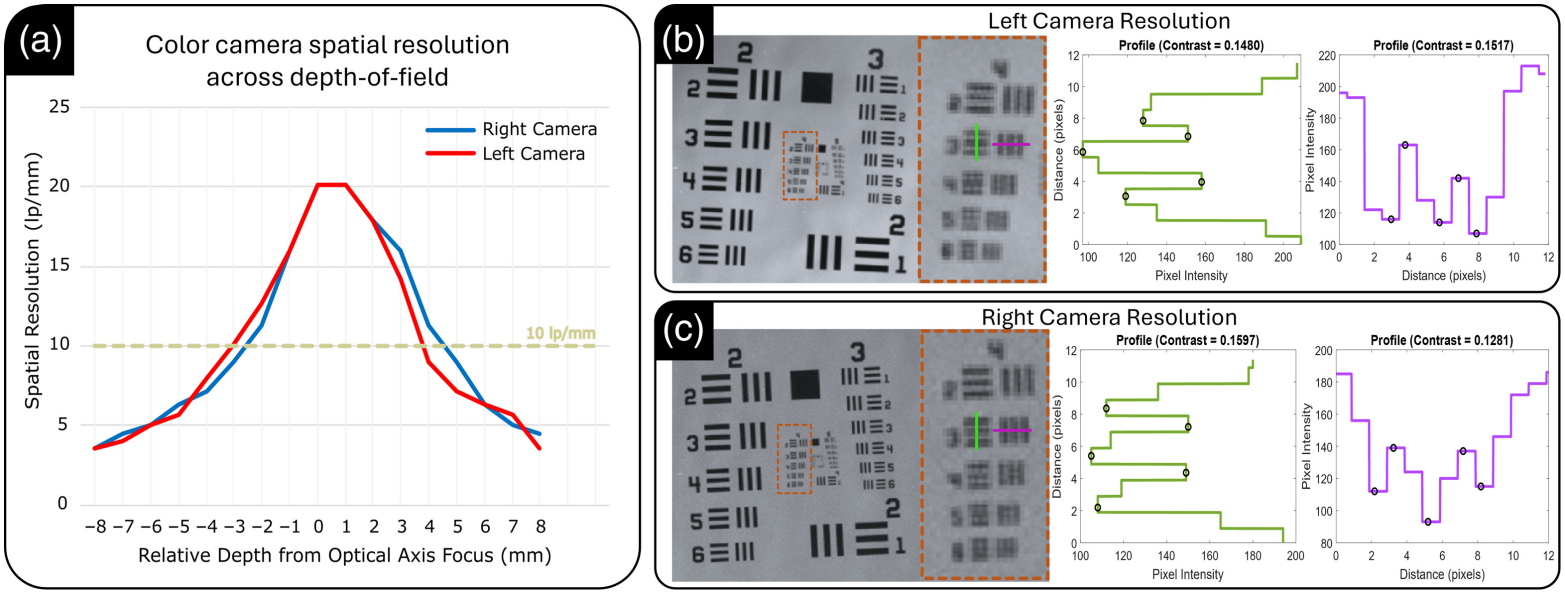
Color camera spatial resolution measurements. (a) Spatial resolution of color cameras as a function of depth of field along the optical axis of the slit illumination subsystem. (b) and (c) USAF 1951 target from left and right color cameras (respectively) with corresponding intensity plots of group 4, element 3.

The color cameras were characterized by evaluating the spatial resolution and depth of field using a USAF 1951 resolution target (Fig. 8). Both cameras achieved a maximum spatial resolution of 20.16  lp/mm, corresponding to a resolvable feature size of 24.80  μm, compared with a theoretical sensor limit of 9.6  μm. Zemax modeling of the lens indicates that the lens is diffraction limited when placed at the optimal distance between the lens and the CMOS sensor. However, resolving power degrades rapidly as the lens is misplaced along the optical axis. We found that shifting the sensor by 1 mm from the lens, theoretical resolution at 10% contrast was reduced to 42.7  lp/mm (see Supplementary Material). Nonetheless, both imaging cameras maintained a resolution of at least 50  μm for 6 mm along the illumination optical axis, enabling the visualization of corneal features across the entire anterior segment.

### 3.4 *In Vivo* Imaging Sessions

**Fig. 9:**
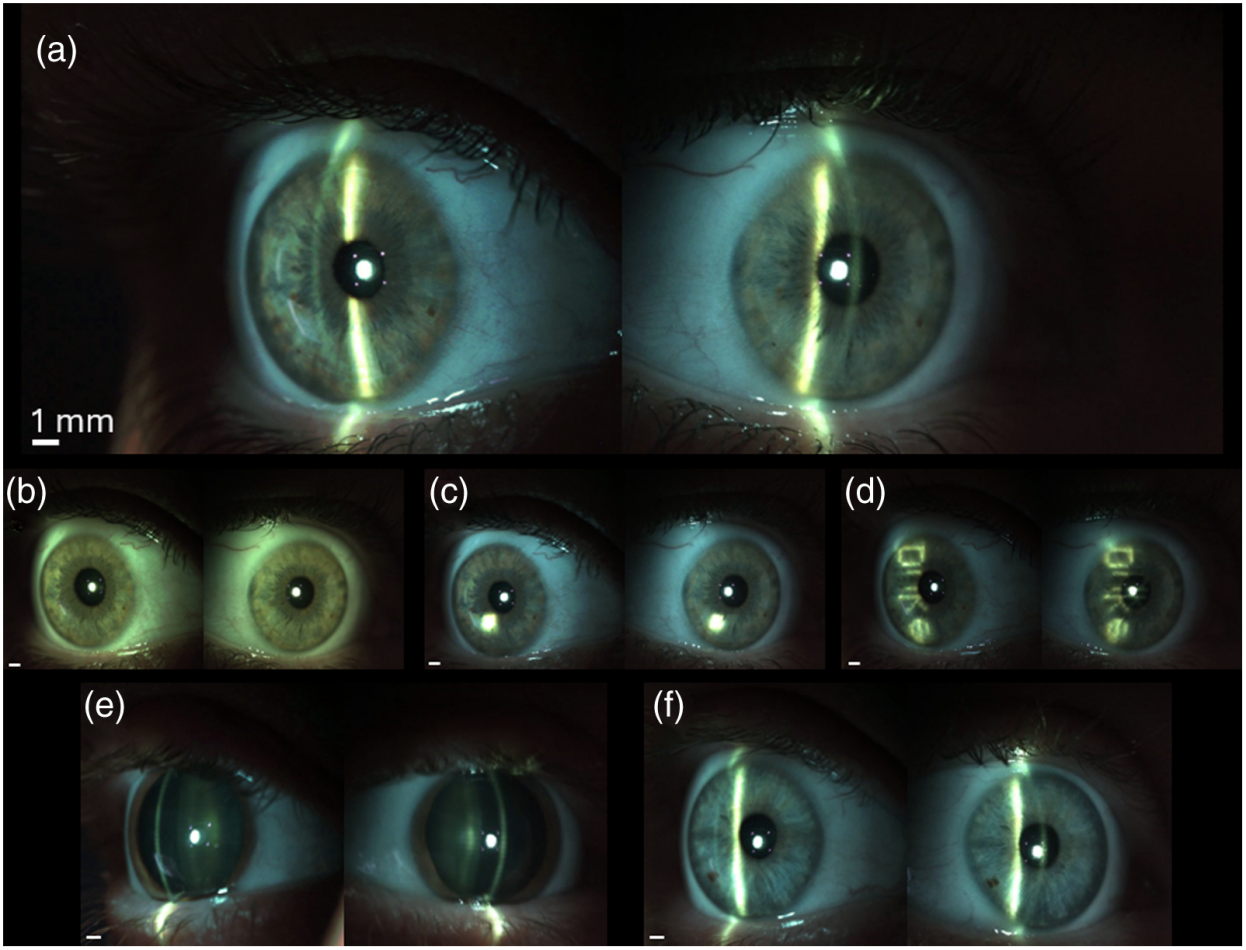
Robotic slit-lamp images from left and right color cameras of subjects’ right eyes during autonomous alignment. Left camera images are histogram matched to the right camera. (a) Slit projected on subject 1’s healthy cornea. (b)–(d) Wide, spot, and “Duke” projected onto subject 1’s cornea. (e) Slit projected on the cornea with a dilated pupil of subject 3. (f) Slit projected on the healthy cornea of subject 2.

**Fig. 10:**
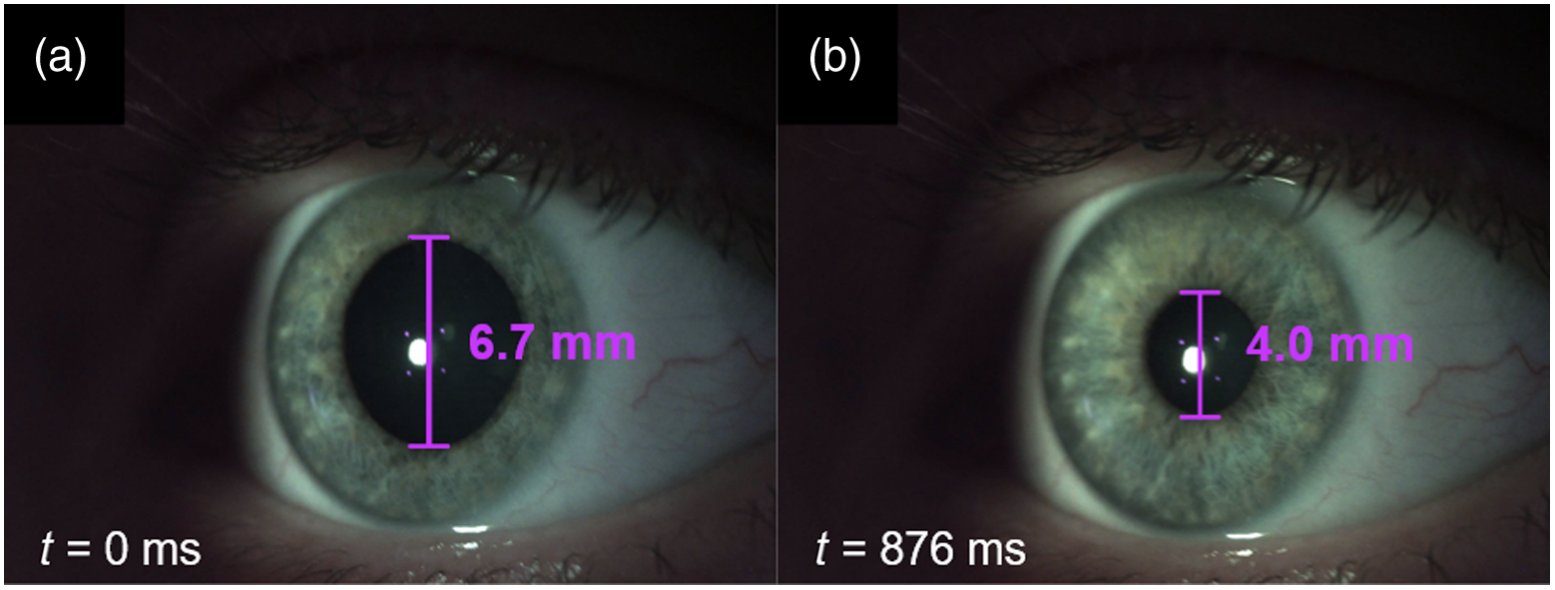
Sequential widefield illumination images demonstrated constrictive pupillary response.

**Fig. 11:**
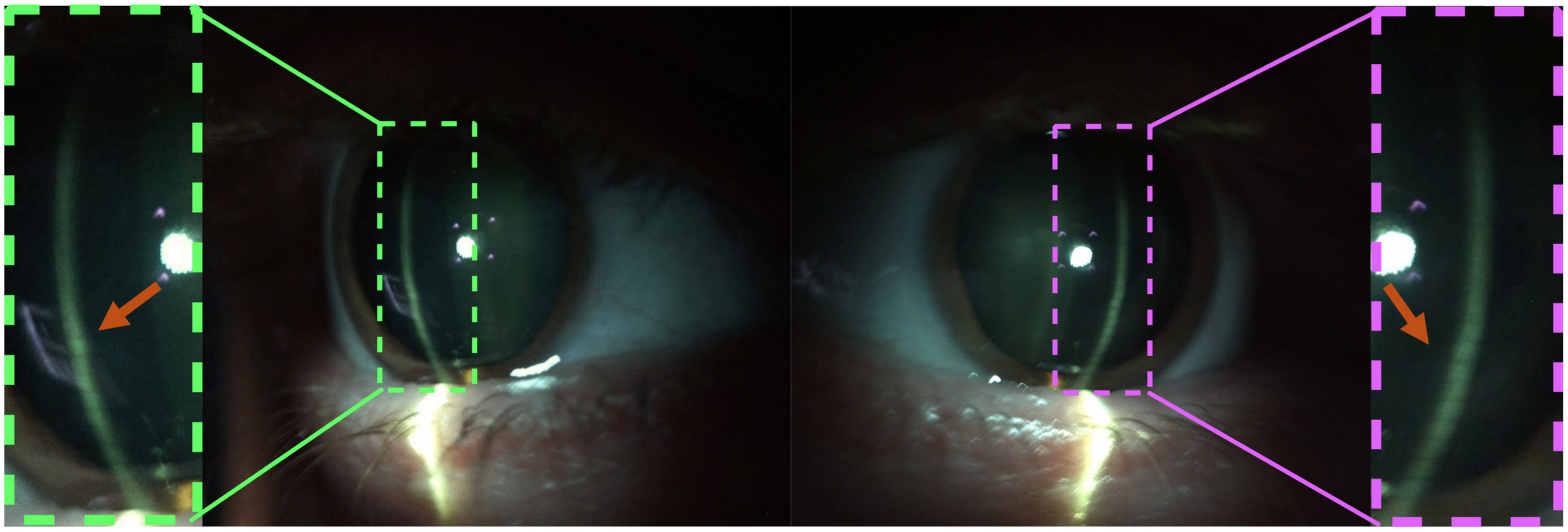
Right (green box) and left (magenta box) camera views of slit projection onto a patient with a subtle corneal scar. The left camera is histogram matched to the right camera. The scar (indicated by the red arrow) scatters more than the surrounding tissue immediately above and below the scar and also caused a subtle flattening or change in the contour of the cornea in the area of the scar. The dark horizontal stripes are an artifact of the LCD screens.

The robotic slit lamp was used to image two healthy subjects and one subject presenting with anterior segment pathology. A stationary slit, spot, full illumination, and the word “Duke” were projected and imaged on each subject’s eyes (Fig. 9). In all color images, the slit pattern illuminates the cornea and iris with Purkinje reflections present from both slit illumination and IR tracking LEDs. Imaging of the healthy subjects demonstrated the system’s ability to project variable illumination patterns onto the cornea, whereas natural constrictive pupillary response created varying tracking conditions. In one healthy subject, we measured pupillary response to a bright stimulus generated by the full illumination pattern. Over 876 ms, we found that the illuminated pupil went from 6.7 to 4.0 mm (Fig. 10), consistent with reported normal pupil size and constriction dynamics in literature.^40^^,^^41^ Figure 11 shows imaging of a clinical patient with a known corneal scar. We were able to identify the scar due to differences in the scattering and anterior profile of the cornea in the area of the scar illuminated by the slit beam, though the stromal cross-section and posterior boundary were less distinct due to the width of the slit.

## 4 Discussion

This work specifically addresses two limitations of conventional slit-lamp imaging: the requirement for mechanical stabilization of the patient and the need for specially trained personnel to operate the device. These limitations are addressed through autonomous robotic alignment and programmable slit illumination, controlled through a simplified user interface. Reducing these constraints is particularly important for environments where ophthalmic expertise is desired but limited, such as urgent care facilities or remote clinics.

Recent developments in portable slit lamps have focused on miniaturization and simplified operation. However, these systems still require fine alignment by the operator, often aided by bracing against the patient by attachments on the device that physically touch the patient. The teleoperated slit lamp described by Nankivil et al.^14^ enables remote imaging by adapting clinically accepted hardware; however, it still requires an operator who understands how to translate conventional slit-lamp control into a virtual interface. In this work, we instead built upon the tracking principles developed for robotic optical coherence tomography systems and adapted these methods specifically for the challenges of visual tracking with active slit illumination. As a result, we utilized the device to see clinically relevant pathology and test the pupillary response in humans. In this study, we anecdotally observed no overt negative volunteer or patient responses while being imaged with our device. Future studies could assess this response quantitatively for the continued development of autonomous medical devices.

Although the presented system demonstrates promising results, there were limitations. The programmable LCD aperture introduces constraints related to pixel size and refresh rate. Commercial slit lamps typically achieve a minimum slit width of ∼0.2  mm, whereas the minimum slit width of our system was 0.36 mm. The magnification of the relayed LCD aperture was chosen to minimize slit width while maintaining sufficient illumination coverage across the iris. The limited refresh rate of the LCD also motivated the inclusion of predictive slit control to maintain alignment under dynamic conditions. In addition, the spacing between LCD pixels produced a grid-like pattern in the image plane. These limitations could likely be mitigated through custom transmissive display fabrication with smaller pixel pitch, higher contrast, and faster refresh rates, which are achievable with current display technologies. For example, recent advances in microfluidic device fabrication have seen use of liquid crystal displays to illuminate features as small as 20  μm.^42^ A commercial transmissive LCD was selected to act as an aperture to enable dynamic control of the slit geometry due to its mechanical and thermal stability, as well as a relatively small footprint compared with mechanical apertures or digital micro-mirror devices (DMDs), despite its relative limitations in contrast and transmission efficiency. The inclusion of a DMD would require a more complex optical layout to account for reflectance angle and magnification of the macroscopic patterns used to illuminate the cornea in slit-lamp photography.

The color imaging subsystem also presents opportunities for improvement. The IMX183 sensor (Sony) provides a relatively large imaging area with small pixels but requires substantial illumination to achieve video-rate acquisition. Custom sensor and lens combinations could improve sensitivity and imaging efficiency while remaining compatible with the mechanical and payload constraints of the robotic platform. Advances in sensor technology, such as single-photon avalanche diode arrays, may increase color image acquisition rates while simultaneously lowering incident power on the eye, improving patient comfort.^43^ We could, alternatively, increase incident power on the eye and subsequently more power on the sensor. The current illumination design limits the light exposure set by group 1 continuous wave instruments in ANSI Z80.36-2021 for high safety margins. Our device, therefore, produces considerably less power than a clinical slit lamp. Adjusting the ANSI category of our illumination from continuous wave to timed or group 1 to group 2 could increase power on the eye while remaining below maximum permissible irradiation limits. In addition, the color imaging resolution deviated from expectation. This could be attributed to the lens working distance being outside of the manufacturer’s design parameters. Furthermore, the Zemax simulation of the lens indicates that small distance errors between the lens and CMOS sensor can impact resolution.

A conventional slit lamp will pivot illumination around a centralized biomicroscope. By contrast, our device maintains a centralized illumination path while positioning the imaging cameras at angular offsets. Despite this unconventional illumination-imaging geometry, providing two simultaneous views combined with dynamic illumination should provide specialists with sufficient information to interpret the acquired ophthalmic data. Recent work from Jiménez-Vilar et al.^44^ utilized stereo imaging offset from a centralized slit scan to create a tomographic map of the cornea for full 3D information from slit-lamp photography, potentially expanding the information gathered from slit exams.

In summary, we have described and shown methods to autonomously align patterned illuminations—like a slit beam, a spot, or arbitrary lettering—on the cornea. Because of this ability to produce and maintain patterned illuminations on the cornea, conceptually other patterns such as the rings used in corneal topography could also be done in the future. The advanced pupil tracking framework used in this work could also support other applications requiring dynamic eye tracking. Overall, we have implemented and demonstrated a prototype for robotically aligned slit-lamp examinations, and the underlying technologies have the potential to support additional types of ocular anterior segment imaging.

## 5 Conclusion

We presented an autonomously aligning robotic slit-lamp capable of imaging the anterior segment without mechanical patient stabilization or specialized operator alignment. Our device utilized vision-based tracking and predictive slit illumination to maintain contactless alignment during imaging. The programmable, patterned illumination compensates for motion with a latency of 86 ms and color imaging has sub-50  μm resolution through the anterior chamber depth. We showed *in vivo* imaging data of healthy and nonhealthy human eyes with varying pupil size. These results show the promise of robotic slit-lamp imaging toward more accessible anterior segment diagnostics.

## Supplementary Material

10.1117/1.JBO.31.12.123304.s01

## Data Availability

The main data supporting the results in this work are available within the paper. The raw data acquired during the study are available from the corresponding author upon reasonable request, subject to approval from the Duke University Health System Institutional Review Board. General robot control code is described in Ref. 21, and the components in which specific modifications were made for this work are available from the corresponding author upon reasonable request.
