# Supplementary material for: Autonomous robotic alignment and dynamic illumination control of an ophthalmic slit lamp for in vivo anterior segment imaging
Source: J Biomed Opt. 2026 Jul 22;31(12):123304. doi: 10.1117/1.JBO.31.12.123304 (PMC13391205; doi:10.1117/1.JBO.31.12.123304)
Supplement: Supplementary file 1 [file JBO_031_123304_SD001.pdf]

1 Supplementary Materials

1.1 Optical system distortion, homogeneity, and contrast calculation

The radial distortion effects of the illumination system were analyzed by following ISO 17850:2015.[1] A CMOS sensor (Sony IMX183) was placed at the image plane of the illumination system and captured images as the projection LCDs in the optical system sequentially activated a single pixel in a 15x15 grid pattern. Each pixel image was then background subtracted, Gaussian filtered, and binarized such that the remaining (largest) connected component could be segmented. The subpixel location of the weighted centroid based on intensity of this connected component could then be used to determine the radial distance from the grid center (H_i in Eqn. 1). To determine the theoretical radial distance for each grid point (H_i^* in Eqn. 1), an idealized 15x15 grid must be created. An affine transform was applied to the points of the idealized grid to account for pixel scaling, shift on the sensor, and minor shear or rotation introduced by an off-axis imaging sensor (Fig. S1A). The local geometric distortion could then be computed across the grid (Fig. 6C).

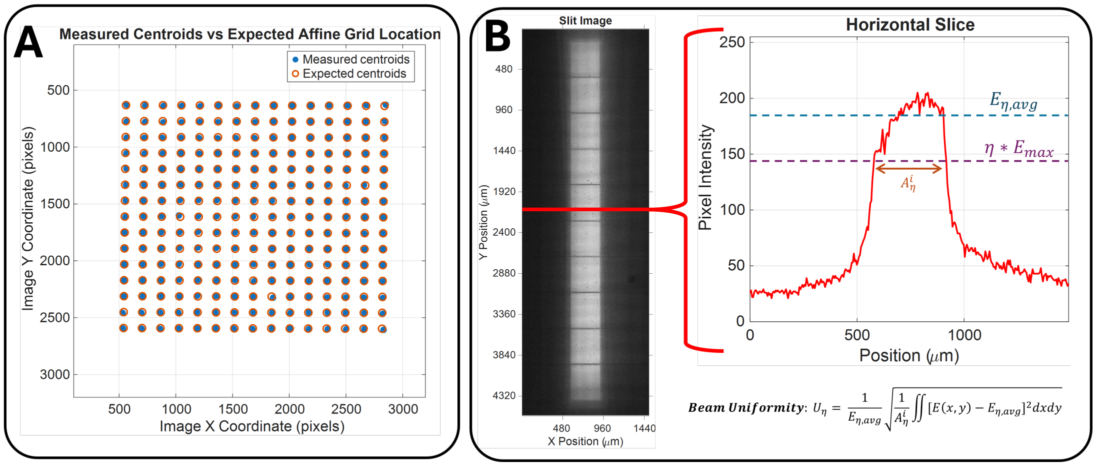

Fig S1 A) The measured and expected centroid locations of a 15x15 grid to measure local geometric distortion. B) An *en-face* image of a slit on a CMOS sensor and an intensity profile to visualize our beam uniformity calculation.

We followed ISO 13694:2018[2] to calculate the beam uniformity of the resulting slit (Fig. S1B). The LCDs were activated such that the system projected a 1x10 slit on a CMOS sensor at the image plane (ON Semi PYTHON 1300). For a given clip level, η, a mask was created for which the image intensity was greater than the threshold $E_{max} * \eta$. The area of the masked region describes A_η. The mean energy density, $E_{\eta,avg}$, was calculated as average pixel density of the masked region. Beam uniformity can then be calculated according to Eqn. 2 by summing and normalizing the root mean square difference between a pixel's energy density from the mean.

Even with all pixels deactivated on the LCDs, some amount of light can be transmitted through the displays and result in undesirable illumination at the image plane. Michelson contrast of the optical system was utilized as a measure of this transmission. First, all LCD pixels were activated such that the system experienced maximal light transmission through the system and the total power, I_{max}, was measured (Thorlabs PM100D and S120C). Then, power was measured again when all LCD pixels were deactivated, I_{min}, such that the system experienced minimal light

transmission. Michelson contrast, C, was then calculated according to Eqn. S1.

$$C = \frac{I_{max} - I_{min}}{I_{max} + I_{min}} \tag{S1}$$

1.2 Imaging optics specifications

Optical imaging components utilized in this system were commercially available (Table S1). The imaging optics were mounted to the robot arm with a combination of commercial optical mounts and custom 3D printed solutions. Additionally, a custom printed circuit board (PCB) with an Arduino Nano Every allowed for real-time communication between our software and the LCDs in the optical path.

Table S1 Imaging optics specifications

**Function**	**Part Number (Quantity) [Vendor]**
Condenser	ACL12708U-A [Thorlabs]
	AC127-050-A (×2) [Thorlabs]
Fly's eye homogenizer	MLA1 (×2) [Thorlabs]
	AC254-100-A [Thorlabs]
	AC254-080-A [Thorlabs]
LCDs	EA DOGM128S-6 (×2) [Display Visions]
Fold mirror	PS911 [Thorlabs]
Final objective	AC254-100-A [Thorlabs]
	AC254-075-A [Thorlabs]

1.3 Color imaging lens simulation

Zemax simulations of the Huygen's modulation transfer function were performed on a black box model provided by the manufacturer of the lens used for color imaging (Edmund Optics 24-472). We performed this simulation at two working distance from the lens to the image plane and multiple distances from the lens to the theoretical camera sensor (Fig. S2). We found that for both scenarios of distance to image plane that the resolving power of the lens was reduced beyond the diffraction limit at a 1mm offset of the sensor to the lens.

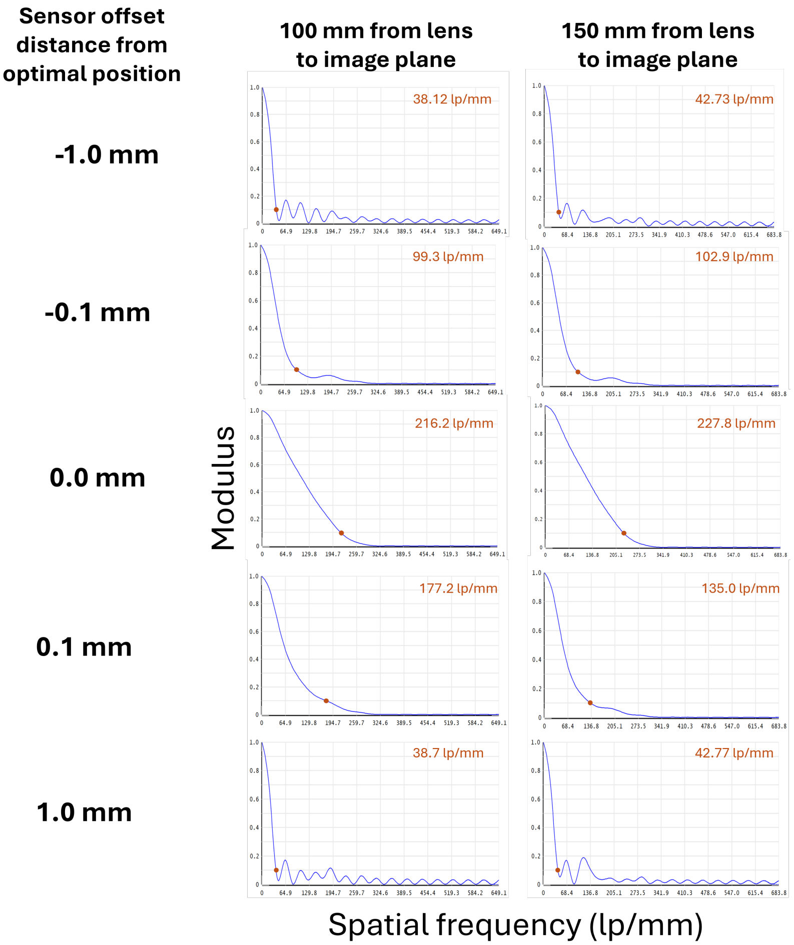

Fig S2 The Huygen's modulation transfer function of multiple variations of the imaging lens' distance to the image plane and distance to the sensor. The portion of the MTF in which contrast is reduced below 10% is marked by an orange dot and the corresponding spatial frequency is noted on the top right of each subplot.

References

1 I. O. for Standardization, "ISO 17850:2015," (2015).
2 International Organization for Standardization, "ISO 13694:2018," (2018).
